# Pre-hospital Delay and Its Reasons in Patients With Acute Myocardial Infarction Presenting to a Primary Percutaneous Coronary Intervention-Capable Center

**DOI:** 10.7759/cureus.12964

**Published:** 2021-01-28

**Authors:** Syed F Mujtaba, Hina Sohail, Jaghat Ram, Muhammad Waqas, Muhammad Hassan, Jawaid A Sial, Khalid Naseeb, Tahir Saghir, Musa Karim

**Affiliations:** 1 Adult Cardiology, National Institute of Cardiovascular Diseases, Karachi, PAK; 2 Cardiology, National Institute of Cardiovascular Diseases, Larkana, PAK; 3 Interventional Cardiology, National Institute of Cardiovascular Diseases, Karachi, PAK; 4 Cardiology, National Institute of Cardiovascular Diseases, Karachi, PAK; 5 Statistics, National Institute of Cardiovascular Diseases, Karachi, PAK

**Keywords:** st-elevation myocardial infarction, pre-hospital delay, primary percutaneous coronary intervention

## Abstract

Objective

This study aimed to assess the duration of pre-hospital delay among ST-Segment Elevation Myocardial Infarction (STEMI) patients and its contributing factors.

Methodology

A cross-sectional study was conducted at Rural Satellite Center in Larkana, Pakistan from May to September 2020. A total of 240 STEMI patients who underwent primary percutaneous coronary intervention (P-PCI) were included. The patients' demographic characteristics, index event characteristics, mode of transportation, misinterpretations, misdiagnoses, and financial problems were recorded. Data were analyzed using SPSS version 22.0 (IBM Corp., Armonk, NY, USA).

Results

The observed pre-hospital time was 120 minutes; 229 (median; interquartile range [IQR]). It was found that 33.3% of patients arrived within one hour of the symptom onset, while 20.4% of patients delayed hospital arrival for more than six hours. The delay rate was highest among patients aged 41 to 65 years. Moreover, delayed admissions were more common among females as compared to males (p=0.008). Among the causes of delay in hospital arrival were misinterpretation, misdiagnosis, and transportation and financial issues. Of these, misdiagnosis significantly influenced the delay rate, i.e., more than 50% of the misdiagnosed patients arrived hospital after six hours of symptom onset (p<0.05).

Conclusion

The P-PCI rural satellite center had a positive impact as the observed pre-hospital delay rate was considerably less as compared to that reported in the existing literature. Moreover, the confounding factors were misdiagnosis and misinterpretations. We need to develop the concept of immediate appropriate help-seeking among patients.

## Introduction

Acute Myocardial Infarction (AMI) remains a medical emergency and a common cause of mortality worldwide. Recommended treatment involves the restoration of blood flow as soon as possible. Reperfusion is achieved either with thrombolytic therapy or percutaneous coronary intervention (PCI) [1,2].

As per the therapeutic guidelines, timely reperfusion is crucial. Therefore, thrombolysis should not be delayed for more than 30 minutes, and PCI to be performed within 90 minutes after arrival at the hospital (door-to-needle time and door-to-balloon time). Primary PCI (P-PCI) is considered superior to thrombolysis [3] as P-PCI has proven to be more efficacious for the cases with prolonged onsets to hospital arrival time. Total ischemia time (TIT), i.e., the time taken from the onset of pain till restoration of perfusion, plays an important role in overall prognosis [4]. Various measures and programs have been developed to cope with pre-hospital delays and enhance care quality [5]. Many developing countries now present the facility of P-PCI capable hospitals with acceptable door-to-balloon time (DBT) [6,7]. Despite all these efforts, no significant reduction in the TIT has been observed until now as the pre-hospital delays, i.e., symptom-to-door time (STDT), remain the major barrier to reducing TIT [8].

For the first time in the province of Sindh (Pakistan), P-PCI-capable satellite centers have been established at strategic locations so that the majority of the patients can reach the facility within two hours by road. This study was aimed to determine the pre-hospital delay in patients with ST-Segment Elevation Myocardial Infarction (STEMI), its contributing factors, and its association with various baseline demographic characteristics. Results of the present study might help in reducing the overall disease burden by notifying the rate of pre-hospital delays and the associated factors. Very few studies on this subject have been conducted in developing countries [6-9].

## Materials and methods

This was a cross-sectional study that was conducted at a Rural Satellite Center (Larkana), Pakistan, from May to September 2020. The study conforms to the Declaration of Helsinki, and the protocol has been approved by the ethical review committee of the National Institute of Cardiovascular Diseases (Ref #: ERC-16/2020). A total of 240 patients fulfilling the inclusion criteria were enrolled in this study after obtaining written informed consent. The inclusion criteria were patients of either gender between the ages of 20-60 years presenting with acute STEMI. In contrast, all patients with prior cardiac-related surgery, chronic kidney diseases (CKD), and congenital heart disease were excluded from the study. Patients who did not undergo P-PCI were also excluded.

Data were collected using a structured questionnaire covering demographic characteristics, predisposing risk factors, care-seeking behaviour of the patients from symptom onset to hospital, reasons for delay presentation, and procedural.

STEMI is defined as chest pain along with ST-segment elevation in two contagious leads on an electrocardiogram (ECG). Patients with new-onset left bundle branch block (LBBB) and confirmed acute blockage of one of the major vessels on angiography were considered as confirmed STEMI cases. Coronary angiography was performed mostly through radial as the preferred route. The procedure was performed within 90 minutes of reaching the hospital, i.e., within 12 hours of the onset of chest pain. The aim was to open the culprit vessels and restore thrombolysis in myocardial infarction (TIMI) 3 flow. The decision regarding stenting and the type of stent was left on the treating consultant. In the case of hemodynamic instability, PCI of the non-culprit artery was undertaken at the consultant's discretion.

Collected data were entered and analyzed using SPSS version 21.0. (IBM Corp., Armonk, NY, USA). Patients were stratified based on symptom onset to hospital arrival time, and ≥ 6 hours of symptom onset to hospital arrival time was considered late arrival. Descriptive statistics such as mean ± standard deviation (SD) or median (interquartile range [IQR]) for continuous measures and frequency and percentages for categorical measures were calculated. Comparison between the two groups, late arrival and timely arrival groups, were made by applying independent sample t-test for continuous response and the Chi-Square test or Fisher's exact test for categorical response variable and significance criteria was p≤0.05.

## Results

**Figure 1:**
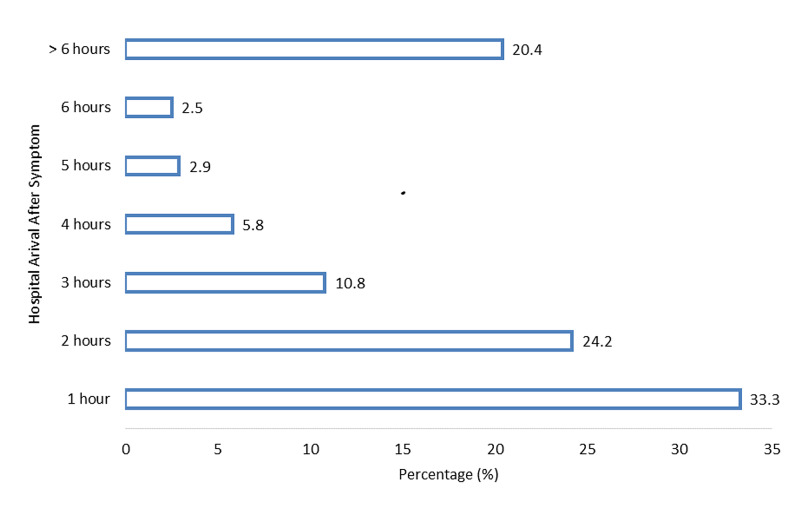
Distribution of patients according to symptom onset of hospital arrival time

A total of 240 patients were included in this study, the majority (77.9%) were males, and the overall mean age was 53.26 ± 10.94 years. The median [IQR] for symptom onset to hospital arrival was 120 [277.5 - 60] minutes. Among these, 20.4% of patients arrived late to the hospital (≥ 6 hours) after symptoms onset. The distribution of patients according to symptom onset of hospital arrival time is presented in Figure 1.

Late arrival was associated with the female gender with a late arrival frequency of 35.8% (19/53) vs. 18.7% (34/188), p=0.008, for female and male patients, respectively. The demographic characteristics of the patients stratified by hospital arrival status are presented in Table 1.

**Table 1:** Demographic characteristics *p-value < 0.05 is considered significant. SD: standard deviation.

Variables		Total N =240	Hospital arrival after symptoms		p-value
			≥ 6 hours (n=54)	< 6 hours (n=186)	
Age (years); mean ± SD		53.26 ± 10.94	54.59 ± 11.28	52.87 ± 10.84	0.310
Age Groups	≤ 40 years	37(15.4)	5(9.3)	32(17.2)	0.340
	41 to 65 years	177(73.8)	42(77.8)	135(72.6)	
	> 65 years	26(10.8)	7(13)	19(10.2)	
Gender	Male	187(77.9)	35(64.8)	152(81.7)	0.008*
	Female	53(22.1)	19(35.2)	34(18.3)	
Marital Status	Married	233(97.1)	54(100)	179(96.2)	0.148
	Unmarried	7(2.9)	-	7(3.8)	
Educational Status	Illiterate	20(8.3)	5(9.3)	15(8.06)	0.894
	Primary	52(21.6)	11(20.3)	41(22.04)	
	Secondary	34(14.1)	8(14.8)	26(13.9)	
	Graduation	40(16.6)	7(2.91)	33(17.7)	
	Not Reported	94(39.2)	-	-	
Economic Condition	Low Class	65(27.0)	15(27.7)	50(26.8)	0.646
	Lower Middle Class	103(42.9)	25(46.29)	78(41.9)	
	Middle Class	51(21.25)	8(14.8)	43(23.1)	
	Upper Middle Class	16(6.6)	4(7.4)	12(6.45)	
	Upper Class	5(2.08)	2(3.7)	3(1.61)	
Residence	Urban	56(23.3)	12(22.2)	44(23.7)	0.826
	Rural	184(76.7)	42(77.8)	142(76.3)	

A significantly higher proportion of late arrival patients had a first impression of non-cardiac chest pain with a frequency of 48.1% (26/54) vs. 32.8% (61/186); p=0.039. First medical contact to the cardiac care center was significantly lower among the late arrival patients than the patients who arrived within six hours of symptom, i.e., 16.7% (9/54) vs. 34.4% (64/186).

Moreover, contact with non-qualified local doctors had a significant role in delaying their presentation to the hospital (p=0.022). The majority of patients (64.8%) who arrived at the hospital in ≥ 6 hours were under the consultation of a local doctor, which significantly affected the hospital arrival rate. Furthermore, the mode of transfer had no significant effect on the pre-hospital delay (p=0.163). Symptom onset and care-seeking behaviour of the patients are summarized in Table 2.

**Table 2:** Symptom onset and care-seeking **Based on patients with the first impression of non-cardiac pain. *p<0.05 is considered significant.

Variables	Total N=240	Hospital arrival after symptoms		p-vlaue
		≥ 6 hours (n=54)	< 6 hours (n=186)	
First impression of symptoms				
Cardiac Pain	153(63.8)	28(51.9)	125(67.2)	0.039*
Non Cardiac pain	87(36.3)	26(48.1)	61(32.8)	
Non-cardiac impression of symptoms**				
Gastric	50(57.5)	14(53.8)	36(59)	0.450
Muscular	13(14.9)	3(11.5)	10(16.4)	
Tension	9(10.3)	2(7.7)	7(11.5)	
Others	15(17.2)	7(26.9)	8(13.1)	
Preferred treatment options				
Home based treatment	83(34.5)	15(27.7)	68(26.5)	0.232
Contacted local doctor	157(65.4)	39(72.2)	118(63.4)	
First medical contact (FMC)				
Local Doctor	113(47.1)	35(64.8)	78(41.9)	0.022*
Local Hospital	29(12.1)	6(11.1)	23(12.4)	
Tertiary Care Hospital	25(10.4)	4(7.4)	21(11.3)	
Cardiac Care Center	73(30.4)	9(16.7)	64(34.4)	
Mode of transfer				
Personal Vehicle	74(30.8)	12(22.2)	62(33.3)	0.163
Taxi	124(51.7)	33(61.1)	91(48.9)	
Ambulance	7(2.9)	-	7(3.8)	
Other	35(14.6)	9(16.7)	26(14)	

Among the major reasons for the pre-hospital delay, misdiagnosis and transportation were significant predictors (p<0.05). No significant effects of misinterpretations and financial issues were observed on the late hospital arrival. Most patients managed to arrive at the hospital in < 6 hours, even with existing misinterpretations and financial issues (p=0.90) (Table 3). 

**Table 3:** Reasons for pre-hospital delay in patients with acute ST-segment myocardial infarction undergoing PPCI *p-value < 0.05 is considered significant. PPCI: Primary Percutaneous Coronary Intervention.

Reasons for delay	Total N=240	Hospital arrival after symptoms		p-value
		≥ 6 hours (n=54)	< 6 hours (n=186)	
Misinterpretation	65(33)	19(29.2)	46(70.7)	0.090
Misdiagnosis	29(21.4)	16(55.1)	13(44.8)	0.000*
Transportation	99(31.1)	13(13.1)	86(86.8)	0.002*
Financial	10(3.9)	1(10)	9(90)	0.299
Others	38(10.7)	5(13.1)	33(86.8)	0.094

## Discussion

The importance of time in the management of myocardial infarction cannot be underestimated. Both mortality and morbidity are directly proportional to the time taken from the onset of symptoms to the start of reperfusion therapy. There are two main treatment modalities of reperfusion therapy: either thrombolysis with pharmacologic drugs or physical removal of clot by the coronary intervention. Thrombolysis is most commonly employed in developing countries. One study from India showed that almost 90% of patients receive thrombolysis than 10% receiving P-PCI [10]. Time is a crucial prognostic marker, whatever treatment option is employed. However, successful reperfusion with thrombolysis is more time-dependent than PCI.

Studies have shown that a major benefit is when patients are perfused within one hour of symptom onset [11,12]. While patients presenting later than 12 hours of symptom onset have no benefit or, in some cases, it exposes them to harm, especially in old-age patients [13,14].

In our study, the observed pre-hospital time (PHT) was 120 minutes (median); 229 (IQR). The data from the CREATE registry of acute coronary syndrome patients involving 89 large hospitals in 10 different regions and cities across India presented a PHT of 300 minutes (137-985) [10]. A more recent study from Bangladesh reported the median pre-hospital delay of nine (IQR 13) hours [15]. Though symptom onset to reperfusion therapy time less than two hours is considered ideal, other studies have shown only 22-44% of patients present to the hospital within two hours of chest pain onset [16-18]. 

In our study, almost 33.3% of AMI patients arrived hospital during the first hour after the symptom onset, 46.25% arrived during two to six hours, and 20.4% presented late, i.e., after six hours of symptom onset. We deliberately selected a cut-off of six hours to label as late, which was used in most studies conducted in developing countries [6]. Our study showed that almost 10% of patients presented even after 12 hours. Other studies showed that almost 10-25% of patients arrived after 12 hours [19-21]. 

Pre-hospital delay is comparatively more as compared to Western countries [22,23]. Short pre-hospital delays in Western countries can be attributed to their well-organized health care system. Early recognition, easy accessibly to hospitals, and early detection are all factors related to a well-organized health care system. One of the major reasons for the pre-hospital delay was the patient's decision time. Almost one-third of the patients misperceived the symptoms. Among them, 50% linked the symptoms to the gastric problem. Similarly, an Indian study also displayed misperception as one of the prominent reasons for the pre-hospital delay, i.e., 24% of their patients considered chest pain of myocardial infarction as gastritis, and 6% perceived it to be a muscular pain [24]. Other studies have also shown that PHT is much reduced when the initial perception of pain is related to a cardiac source rather than a non-cardiac source of pain [7,9]. 

Ischemic heart disease is commonly considered a disease of urban society. As most of our study population was from rural areas, the cardiac origin of pain might have been neglected due to lack of knowledge as misperceived as gastric pain, which is commonly treated with home remedies. Almost 30% of the enrolled patients initially took some home-based remedy for symptomatic relief, resulting in delayed PHT.

The current study showed that approximately 79% of the total pre-hospital delays were due to patient-related factors, and only 21% of the delays were due to transportation. They also showed that a good number of hours are wasted prior to physician consultation [25]. One solution is large scale programs of awareness of heart attack symptoms and signs. The population should be made aware to immediately contact the doctor with an ECG facility to diagnose AMI to initiate prompt management. But previously, large scale education programs for awareness about myocardial infarction symptoms have not proved to be significantly decreasing the PHT.

A recent systematic review of 10 studies aiming to reduce pre-hospital delay times concluded that there was little evidence that public education interventions reduced pre-hospital delay [26]. Similarly, the REACT study carried out from 1995 to 1997 in 20 American cities concluded that although persons living in the target areas of the public information campaigns were demonstrably better informed about the subject afterwards; however, the PHTs in the target areas were not shortened significantly in comparison to control areas [27].

One justification can be that these studies were conducted in developed nations where education and awareness level was already optimum, and similar programs would likely bear fruitful results in our population with low literacy. Transportation was also a major reason for late arrival. Our study population mostly consisted of rural areas. This area has not a well-established system of roads. Transport means are mostly private owned or taxis. Finding a taxi is sometimes difficult in rural areas. The widespread use of mobile phones has made somewhat things better. A reason for delays due to transportation is the lack of an organized ambulance service. Practically no organized ambulance service is available at the government level. Therefore the patient has to be carried to the hospital via self-transportation.

The third reason was misdiagnosis at the primary consultation point. Due to the lack of a well-functioning primary health care system, only 10% went to a local hospital in our study. Almost 50% of the patients went to a local doctor. These local doctors are mostly medical graduates. They work as family physicians. Most of the time, they are not well trained in cardiology or lack basic equipment like ECG. Therefore the misdiagnosis rate is high. Thus, there is a strong need for these primary health care providers to identify signs and symptoms of myocardial infarction and be equipped with an ECG facility.

## Conclusions

In conclusion, the enrolled STEMI patients presented to the Rural Satellite Center in Larkana, Pakistan, displayed relatively short pre-hospital delay time as compared to other reports from developing countries. Symptom impressions, contact with non-qualified practitioners and misdiagnosis had a significant role in delaying the hospital arrival. It is crucial to develop an adequate health care delivery system to relay effective and timely diagnosis and treatment. Moreover, public awareness is also very important to reduce pre-hospital delay.
